# Occurrence and quantification of Shiga toxin-producing *Escherichia coli* from food matrices

**DOI:** 10.14202/vetworld.2018.104-111

**Published:** 2018-02-03

**Authors:** C. Sethulekshmi, C. Latha, C. J. Anu

**Affiliations:** Department of Veterinary Public Health, College of Veterinary and Animal Sciences, Mannuthy, Thrissur, Kerala, India

**Keywords:** food matrices, occurrence, polymerase chain reaction, real-time quantitative polymerase chain reaction, Shiga toxigenic *Escherichia coli*

## Abstract

**Aim:**

The objective of the study was to detect Shiga toxin-producing *Escherichia coli* (STEC) and develop a quantitative polymerase chain reaction (qPCR) assay to quantify the bacterial DNA present in different food matrices.

**Materials and Methods:**

A total of 758 samples were collected during a period from January 2015 to December 2016 from Kozhikode, Thrissur, and Alappuzha districts of Kerala. The samples consisted of raw milk (135), pasteurized milk (100), beef (132), buffalo meat (130), chevon (104), beef kheema (115), and beef sausage (42). All the samples collected were subjected to isolation and identification of STEC by conventional culture technique. Confirmation of virulence genes was carried out using PCR. For the quantification of STEC in different food matrices, a qPCR was standardized against *stx*1 gene of STEC by the construction of standard curve using SYBR green chemistry.

**Results:**

The overall occurrence of STEC in raw milk (n=135), beef (n=132), buffalo meat (n=130), chevon (n=104), and beef kheema (n=115) samples collected from Kozhikode, Thrissur, and Alappuzha districts of Kerala was 19.26%, 41.6%, 16.92%, 28.85%, and 41.74%, respectively. PCR revealed the presence of *stx* 1 and *stx* 2 genes in 88.46 and 83.64 and 30.77 and 40.00% of STEC isolates from raw milk and beef samples, respectively, while 100% of the STEC isolates from buffalo beef and beef kheema samples carried *stx* 1 gene. Real-time qPCR assay was used to quantify the bacterial cells present in different food matrices. The standard curve was developed, and the slopes, intercept, and R^2^ of linear regression curves were −3.10, 34.24, and 0.99, respectively.

**Conclusion:**

The considerably high occurrence of STEC in the study confirms the importance of foods of animal origin as a vehicle of infection to humans. In the present study, on comparing the overall occurrence of STEC, the highest percentage of occurrence was reported in beef kheema samples. The study shows the need for rigid food safety measures to combat the potential pathogenic effects of harmful bacteria throughout the production chain from production to consumption.

## Introduction

In recent years, *Escherichia coli* has become an organism of public health significance due to its association with life-threatening diseases in human beings. Among *E. coli*, Shiga toxigenic *E. coli* (STEC), especially the serotype O157:H7, is an emerging foodborne pathogen capable of causing potentially fatal illnesses such as hemorrhagic colitis, hemolytic uremic syndrome, and thrombocytopenic purpura in man [1,2]. The infection occurs in humans through the acquisition of the bacteria through consumption of contaminated food. Raw or undercooked foods from animal origin have been recognized as the most important vehicle for transmission of STEC [3-5]. Nevertheless, lack of knowledge on good hygienic practices and the prevailing unsanitary conditions in food processing environment can cause cross-contamination of food [6].

Conventional methods used for isolation and identification of *E. coli* are ineffective for the isolation of STEC, for which specific methods are required [7]. Thus detection of STEC demands one or more selective enrichment steps followed by plating onto a selective agar [8]. Hence, for the effective isolation of STEC, the biochemical characteristics such as β-glucuronidase activity and the inability to rapidly ferment sorbitol have been exploited, and various selective and differential media have been developed [9]. As these methods are time-consuming, analytical methods based on bacterial nucleic acids are being widely used [10].

The STEC strains isolated from cattle, food, and other animal sources have various virulence profiles, and to assess the potential virulence of STEC isolates from these sources, it is important to examine them for the presence of virulence genes. The virulence of STEC strains is mainly associated with their ability to damage intestinal epithelial cells and produce Shiga toxins *stx*1 and or *stx* 2. The polymerase chain reaction (PCR) is widely used for the detection of virulence factors and is known to be a sensitive and specific method. Development of quantification methods for bacterial cells in food matrices aids in the screening of food samples for the presence of potential pathogens and aids in detecting the level of contamination. The accurate quantification of *stx* 1 gene carrying STEC can be accomplished by real-time quantitative qPCR method [7,11]. The study aims to detect STEC and to develop a qPCR assay to quantify the bacterial DNA present in different food matrices.

## Materials and Methods

### Ethical approval

Ethical approval was not necessary for this study. However, samples were collected as per standard collection procedure described by Taylor *et al*. [12].

### Bacterial strains

Reference culture of *E. coli* (MTCC 3221) was procured from the Institute Of Microbial Technology, Chandigarh, India. The STEC culture maintained in the repository of the Department of Veterinary Public Health was used for the study. The cultures were stored in nutrient broth (Difco Laboratories, Detroit, MI, USA) containing 10% sterile glycerol at 70°C. Cultures were grown overnight (18 h) in Luria broth (Difco) at 37°C as proposed by Yang *et al*. [13].

### Collection of samples

A total of 758 samples were collected during a period from January 2015 to December 2016 from Kozhikode, Thrissur, and Alappuzha districts of Kerala. The samples consisted of raw milk (135), pasteurized milk (100), beef (132), buffalo meat (130), chevon (104), beef kheema (115), and beef sausage (42). The aseptically collected samples were brought to the laboratory under the refrigerated condition and processed for the analysis.

### Isolation of STEC by conventional method

All the samples collected were subjected to isolation and identification of STEC by conventional culture technique. The samples were transferred to TSB and homogenized. Isolation and identification of STEC from collected samples were carried out by pre-enrichment and selective enrichment followed by selective plating as described by Meng *et al*. [14]. The isolates were further confirmed by plating onto 4-methylumbelliferyl-beta-D-glucuronide (MUG EC) agar [15]. The colonies showing characteristics neutral gray to colorless colonies with smoky centers on CT-SMAC agar were then subjected to primary and secondary biochemical identification test. Then, the identification of virulence genes was carried out using PCR [16].

### Molecular characterization of STEC

#### Extraction of DNA from bacterial culture

The DNA was extracted from individual bacterial cells using the QIAamp DNA Mini Kit (Qiagen), according to the manufacturer’s instructions. The bacterial cells from a 250 μl aliquot of culture were obtained by centrifugation (14000 g, 2 min, 18±20°C) and resuspended in 45 μl 10 mM phosphate buffer, pH 6±7, before freezing at −20°C. The frozen cells were then heated in a boiling water bath for 10 min.

The calculation of target DNA copies was done with the following equation: Number of copies (molecules)=X ng×6.0221×10^23^ molecules per mole/(N×660 g/mole)×10−^9^ ng/g), where X is the mass in nanogram, and N is the length of the genomic DNA [17].

### Primers used for the identification of STEC stx 1, stx 2, eae A, and hly A genes

Four oligonucleotide primers, targeting the Shiga toxin gene 1 (*stx*1), Shiga toxin gene 2 (*stx*2), *Enteroeffacement* gene A (*eae*A), and *Enterohemolysin* A (*hly*A) was used in the study for the detection of STEC (Table-1) [16,18,19]**.

**Table-1:** Primers used for the identification of STEC *eae*A, *st*χ 1, *st*χ 2, and *hly* A.

Genes	Primer	Length	Primer sequence	Size of amplicon (bp)	References
*eae A*	F	20	5’-CTGAACGGCG ATTACGCGAA-3’	917	[18]
	R	18	5’-CCAGACGAT ACGATCCAG -3’		
*Stχ*1	F	23	5’ATAAATCGCCAT TCGTTGACTAC -3’	180	[16]
	R	21	5’-AGAACGCCCA CTGAGATCATC -3’		
*Stχ*2	F	21	5’-GGCACTGTCT GAAACTGCTCC-3’	255	[18]
	R	22	5’-TCGCCAGTTAT CTGACATTCTG -3’		
*hly*A	F	20	5’-TGGTGCAGCA GAAAAAGTTG-3’	244	[19]
	R	20	5’-ATCCTCTCC TTCCCGTTGTT-3’		

### Setting up of PCR

The PCR reaction mixture was a total of 25 µL containing 2.5 µL DNA extract, 2.5 µL of 10× buffer (Sigma^®^), 2.0 µL of 10 mMdNTPs mix (Fermentas^®^), 1.0 µL of 20 µM primers, 2.5 µL of 25 mM MgCl_2_, and 1 U of Taq Polymerase (Fermentas^®^). PCR amplification was performed in an automated thermal cycler (Eppendorf Master Cycler, Germany) with initial denaturation at 95°C for 7min. This was followed by 40 cycles of denaturation at 95°C for 1 min; annealing at 56°C for *stx*1 for 40 s and 61.6°C for *stx*2 for 1 min; and 61°C for 1 min for *eae*A and *hly*A followed by extension at 72°C for 10 min. 10 µL of the PCR product was electrophoresed in an agarose gel (1.5%) containing 5 µL of 10 mg/ml ethidium bromide at 80 V for 60 min. 50 bp DNA marker (Fermentas^®^) was used as molecular size marker. DNA amplifications were examined and results documented using Gel Documentation System (Syngene^®^).

### Real-time PCR assay for quantification of STEC

The standardization of qPCR for gene *stx*1 of STEC was achieved by construction of standard curve using SYBR green chemistry. Standard curves were generated for quantification purposes using genomic DNA (carrying *stx*1 gene). Serial dilutions ranging from 10^1^ to 10^7^ were prepared using 1 µL of the target DNA concentration (copies of DNA) assuming that one copy of DNA is equal to one colony-forming unit (CFU). The same primer pairs used in the traditional PCR were used to construct a standard curve as well as to ascertain the possible detection limit.

The maxima SYBR Green qPCR master mix (2X) with ROX was procured from Thermo Scientific India. The preparation of master mix included all reaction components except template DNA. The reactions were performed (Applied Biosystems, Sweden) in a final volume of 12.5 µL reaction mixture using Maxima SYBR Green/ROX qPCR 6.25 µL and forward and reverse primer (10 pM/µl) 0.5 µL each. The reaction conditions for amplification of DNA were 95°C for 10 min, 40 cycles of 95°C for 15 s, and 56°C for 40 s. The reaction included a positive control and a non-template control used as a negative control to check possible reagent contamination.

Data analysis made use of Sequence Detection Software version 1.6.3 supplied by Applied Biosystems. The standard curve was calculated by plotting the log of starting a quantity of DNA copy numbers against cycle threshold (Ct) values generated. A melt curve was generated to verify the product by its specific melting temperature.

### Statistical analysis

The data obtained were subjected to statistical analysis using the SPSS version 21.0. The Fisher’s exact test was used to assess the significance of the occurrence of STEC within the same sample between different sources within the same district as well as between districts.

Cochran’s Q-test was used to compare the occurrence of virulence genes in STEC organisms. This test was used to determine whether there was any statistically significant difference between *stx*1, *stx*2, *hly*A, and *eae*A genes in STEC isolates.

## Results

### Occurrence of STEC by conventional method

The highest occurrence (37.50%) of STEC was detected in raw milk samples collected from Alappuzha district. However, STEC was isolated from 12% and 11.11% of samples belonging to Thrissur and Kozhikode districts. An overall occurrence of the organism was observed in 19.26% of raw milk samples from the three districts. Statistical analysis by Fisher’s exact test showed a significant difference (p≤0.05) in the occurrence of STEC in raw milk samples between districts. The samples collected from Kozhikode and Thrissur differed significantly from those collected from Alappuzha district (Table-2). STEC was not detected in any of the pasteurized milk samples collected from the three districts.

**Table-2:** Occurrence of STEC in different food matrices.

Meat samples	Districts					

	Kozhikode		Thrissur		Alappuzha	
		
	Total samples	STEC-positive samples n (%)	Total samples	STEC-positive samples n (%)	Total samples	STEC-positive samples n (%)
Raw milk	45	5 (11.11^a^)	50	6 (12.00^a^)	40	15 (37.50^b^)
Beef	44	14 (31.82^a^)	58	25 (43.10^a^)	30	16 (53.33^a^)
Buffalo meat	40	7 (17.50^a, b^)	46	13 (28.26^b^)	44	2 (4.55a)
Chevon	30	5 (16.67^a^)	42	14 (33.33^a^)	32	11 (34.38^a^)
Beef Kheema	40	16 (40.00^a^)	40	17 (42.50^a^)	35	15 (42.86^a^)
Beef Sausage	12	0 (0.00^a^)	14	0 (0.00a)	16	0 (0.00^a^)
Total	166	42 (25.30)	200	69 (34.50)	157	44 (28.03)

Figures bearing the same superscript in the same row do not differ significantly (p≤0.05). STEC=Shiga toxin-producing *Escherichia coli*

Occurrence of STEC was highest in beef samples collected from Alappuzha (53.33%). Samples from Kozhikode and Thrissur were contaminated with the organism at the rate of 31.82% and 43.10%. The STEC was present in 55 samples of a total 132 beef samples examined. Occurrence of STEC was high in buffalo meat samples collected from Thrissur. Thirteen samples revealed the presence of the organism. However, seven and two STEC isolates were recovered from samples from Kozhikode and Alappuzha, respectively. An overall occurrence of STEC was detected in 16.92% of samples. The statistical analysis by Fisher’s exact test revealed that, in buffalo meat, the occurrence of STEC in Thrissur significantly differed (p≤0.05) from that of Alappuzha district. The STEC was isolated from more than 30% of chevon samples from Thrissur and Alappuzha. The 16.67% of chevon samples from Kozhikode showed the presence of the organism. An overall occurrence of 28.85% of STEC was reported in chevon samples from the three districts. The occurrence of STEC organism in beef kheema was in the order of 16, 17, and 15 samples, respectively, from Kozhikode, Thrissur, and Alappuzha districts. A total of 41.74% samples showed the presence of the organism. The beef sausage samples examined did not reveal the presence of STEC in any of the samples examined.

### Occurrence of STEC virulence genes in different samples

**Figure-1:**
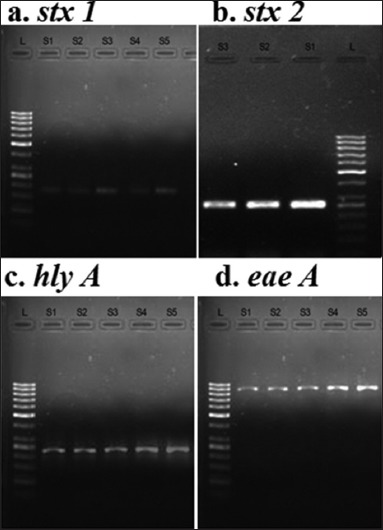
PCR results for virulence genes of EHEC.

The STEC detected in 26 raw milk samples by conventional culture method was subjected to PCR for the confirmation. All the 26 positive isolates were identified to carry either of *stx* 1, *stx* 2, or *hly*A genes using PCR. The gene *stx* 1 was detected in 23 isolates, while eight of the isolates carried *stx* 2 gene, of which five isolates carried both *stx* 1 and *stx* 2 genes. The *hly*A genes were detected in combination with two and one isolates carrying *stx* 1 and *stx* 2 genes, respectively. However, *eae*A gene was not be detected in any of the positive isolates. The statistical analysis was done using Cochran’s Q-test which revealed that the occurrence of gene *stx* 1 was significantly different (p≤0.05) from that of all other genes in positive isolates from raw milk samples. All of the positive isolates derived from beef, buffalo meat, chevon, and beef kheema were identified to carry either of *stx* 1, *stx* 2*, eae*A, and *hly*A genes using PCR (Figure-1). The gene *stx* 1 was identified in cent % of positive isolates from buffalo meat and beef kheema samples (Table-3). While STEC isolated from beef and chevon samples harbored *stx* 1 gene in 83.64% and 83.33% of the isolates. The *stx* 2 gene was identified in 40% and 26.67% STEC isolates in beef and chevon samples, respectively. The STEC isolates from meat and meat product samples also harbored genes *hly*A and *eae*A in combination with either of *stx* 1 or *stx* 2 or both genes. A high % (12.50) of *eae*A gene carrying isolates was found in beef kheema, in combination with *stx* 1 gene. The statistical analysis using Cochran’ Q-test which revealed that the occurrence of gene *stx* 1 was significantly different (p≤0.05) from that of all other genes in all the samples. The isolates from beef samples showed a significant difference in the occurrence of *stx* 1 genes to that of *stx* 2, *hly*A, and *eae*A genes, but no significant difference was noticed between *stx* 2 and *hly*A genes. However, *eae*A gene differed significantly from that of *stx* 1 and *stx* 2 genes but not with that of *hly* A genes.

**Table-3:** Occurrence of STEC virulence genes (%) in different samples.

Virulence genes	Raw milk	Beef	Buffalo meat	Chevon	Meat products
*stχ* 1	88.46^a^	83.64^a^	100.00^a^	83.33^a^	100^a^
*stχ* 2	30.77^b^	40.00^b^	27.27^b^	26.67^b^	41.67^b^
*eae* A	0^b^	7.27^c^	4.54^b^	6.67^b^	12.5^b^
*hly*A	11.54^b^	23.64^c,b^	22.73^b^	20.00^b^	14.58^b^
Total isolate	26	55	22	30	48

Figures bearing the same superscript within the same column do not differ significantly (p≤0.05). STEC=Shiga toxin-producing *Escherichia coli*

### Quantitation of STEC by real-time PCR

**Figure-2:**
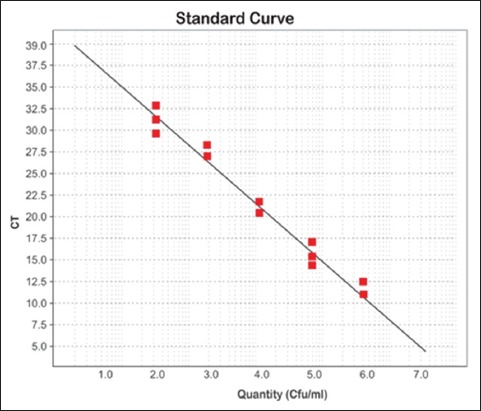
Standard curve of Enterohaemorrhagic *E. coli*.

**Figure-3:**
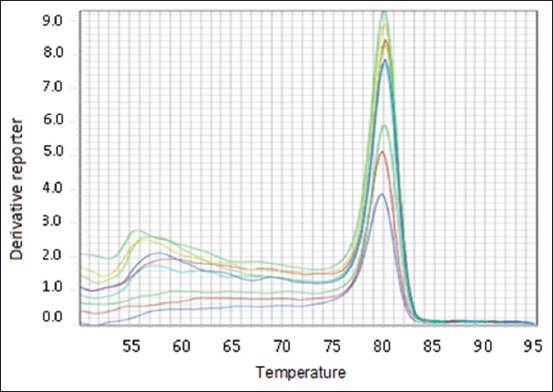
Melt curve for EHEC.

**Figure-4:**
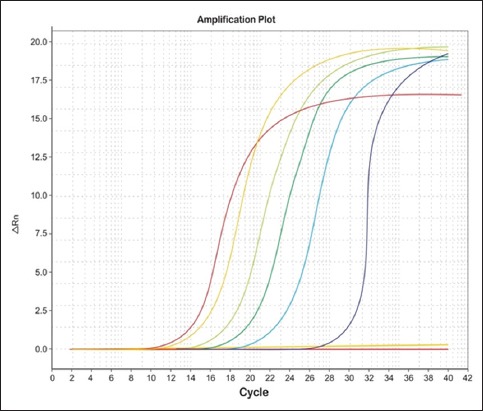
Amplification plot of EHEC.

One microliter of DNA containing 4.09 × 10^8^ copies of *stx* 1 gene was subjected to serial dilution ranging from 10^1^ to 10^7^. Ct values increased at each dilution with the corresponding decrease in target DNA concentration. This clearly demonstrated the validity of the assay, showing that quantification of target DNA is possible. Amplification plot generated corresponding to the standard curve showed a linear correlation between log _10_ copy numbers and Ct with slope values and R^2^ values (Figures-2-4). The slopes, intercept, and R^2^ values of linear regression curves were −3.10, 34.24, and 0.99, respectively. The melting temperature was determined at 79.1°C.

The representative samples from different sources which were positive for STEC by PCR were quantified using real-time PCR (Table-4). The concentration of DNA copies in unknown samples was estimated from the standard curve assuming that one copy of DNA is one CFU. The Ct values corresponding to the log of copy numbers gives the unknown sample concentration.

**Table-4:** Result of quantitative detection of STEC by real-time PCR.

Sources	Mean count±SD (CFU/ml)
Raw milk	1.0×10^1^±0.18
Beef	3.2×10^4^±0.12
Buffalo meat	2.7×10^4^±0.26
Chevon	3.0×10^2^±0.13
Beef kheema	3.4×10^4^±0.16

STEC=Shiga toxin-producing *Escherichia coli*, PCR=Polymerase chain reaction, CFU=Colony-forming unit, SD=Standard deviation

## Discussion

Globally, STEC is regarded as the major cause of foodborne disease outbreaks. Consumption of contaminated foods of animal origin plays an important role in STEC transmission as ruminants are known reservoirs for STEC.

In the present study, maximum STEC contamination in raw milk samples was observed in the samples collected from Alappuzha district, where STEC was recovered from 37.50% of samples. Statistical analysis showed a significant difference in the occurrence of STEC in Alappuzha district from rest of the districts. Various studies showed the prevalence of EHEC in raw milk samples at different levels. A considerable number of isolation of EHEC in milk samples were made by many investigators. The milk samples which were positive for the organisms in the earlier studies were from individual as well as from bulk milk samples. This means that the milk is contaminated at the production level itself. In the present study, individual milk samples were collected from the milk societies before they were pooled directly from the farmer. Hence, the results indicate that the hygienic condition at the production level is far below. The contaminated udder, environment, or the milkers might have contributed the organisms to the milk. Similar results as of the present study were obtained by Mohammadi *et al*. [20] who had reported an overall prevalence of 17.47% for STEC in 206 raw milk samples collected from Kermanshah, Iran. Fecal contamination due to poor hygiene is a potential risk factor for the presence of pathogenic organisms in milk. The considerably high occurrence of STEC in the study confirms the importance of raw milk as a vehicle of infection to human.

The organism was not be isolated from any of the pasteurized milk samples examined. The previous studies carried out by Junior *et al*. [21] and Hoffmann *et al*. [22] also reported that the pasteurized milk was free from STEC. The above findings revealed that pasteurization aids in the effective elimination of pathogen from raw milk. Hence, consumption of pasteurized milk is a safe practice to prevent milk-borne STEC outbreaks.

On comparing the overall occurrence of STEC, highest % of occurrence was reported in beef kheema samples. The STEC in these samples had been detected at a rate of 41.74% of samples. Temelli *et al*. [23] reported a similar occurrence rate to that of the present study in ground beef samples examined. Fantelli and Stephan [24] and Maktabi *et al*. [25] reported a lower occurrence rate of 2.3% and 1.5% than the present study from minced beef samples. Next to beef kheema, beef samples represented the highest % in the occurrence of STEC. The beef samples were contaminated with STEC at a rate of 41.67%, followed by chevon (28.84%) and buffalo meat (16.92%) samples. Arthur *et al*. [26] recovered STEC from 43.4% of pre-eviscerated beef carcasses. The comparatively higher occurrence rate of EHEC obtained in the study could be attributed to the asymptomatic carriage of EHEC in the farm animals and the fecal contamination of the meat during slaughter. Moreover, the animals are slaughtered in abattoirs and sometimes in the backyards without observing hygienic practices. Butchers and meat sellers pay little attention to their personal hygiene. The cutting table and the knives used were not sanitized properly. Moreover, meat was sold in open markets. Rahimi *et al*. [27] reported the presence of STEC in 8.2%, 5.3%, and 1.3% of beef, water buffalo, and chevon samples, respectively, which is lower than the present study. A higher occurrence than the present study was reported by Sinha *et al*. [28] who had isolated STEC from 37.33% of buffalo meat samples belonging to Gujarat, India. The presence of STEC has been reported previously in goat meats [29-31]. Momtaz *et al*. [32] reported an isolation of STEC from 18.66% of ruminant meat samples. The lack of hygienic slaughterhouses along with unhygienic post slaughter facilities might have contributed to the relatively high occurrence of STEC in buffalo beef samples.

The detection of virulence genes in the STEC isolates is a clear confirmation of result obtained in the conventional culture method. Among the STEC isolates from various food matrices, *stx* 1 gene was more predominantly found than the *stx* 2 gene, followed by *hly* A and *eae*A genes. Similar findings were reported in studies carried out by Rashid *et al*. [33] and Ranjbar *et al*. [34] where *stx* 1 was more frequently detected than *stx*2 and *ehly* genes. The presence of virulence genes in the isolates indicates that the STEC organism present in the meat and meat products were of highly pathogenic in nature and can cause a serious public health hazard.

In the present study, real-time PCR was standardized by creating standard curves for *stx*1 gene. The target gene used in the study was previously used by Ibekwe *et al*. [35] for quantification of *E. coli* O157:H7 in environmental samples. The real-time PCR has been used to quantify STEC organisms from food and environmental samples by Bellin *et al*. [36]. For detection of STEC from food, Hara-Kudo *et al*. [37] found that the real-time PCR was more sensitive than the conventional culture technique. The Ct is the number of cycles required for the fluorescent signal to cross the threshold. The Ct values are inversely proportional to the amount of target nucleic acid in the sample [38]. In the present study also, Ct values increased at each dilution, demonstrating the validity of the assay as the target DNA concentration decreased with each cycle and showed that the quantification of target DNA is possible.

Quantitative real-time PCR analysis of the raw milk, beef, buffalo beef, chevon, and beef kheema samples for the *stx* genes with the standard curves revealed linearity between the Ct values and the starting quantity of DNA copy numbers. These standard curves were used for estimating the numbers of STEC in the samples. The ability to quantify STEC in different samples will aid in developing models of pathogen transport in the environment and subsequently assist in the development of risk assessment strategy. Bono *et al*. [39] used real-time PCR to detect STEC. The average Ct of the *E. coli* O157 isolates was 27 cycles. They reported that 87 of the 102 non-*E. coli* O157 isolates from beef samples did not amplify after 45 cycles and thus had no Ct values. According to Ibekwe *et al*. [35], the Ct values showed a linear relationship with the number of *E. coli* O157:H7 organisms added, indicating a direct correlation between the Ct and the number of *E. coli* O157:H7 CFU per g of feces. The above results indicate that the real-time qPCR assay is useful in screening the foods of animal origin carrying Shiga toxin genes.

## Conclusion

STEC is an emerging foodborne pathogen of public health importance that hit the food industry over the past two decades. This study was performed with the objective of assessing the occurrence of STEC in different samples from different sources, screening the positive isolates for the presence of virulence genes, and quantifying the organism in the positive samples. The results of the study revealed the ubiquitous nature of STEC and its widespread presence in the foods of animal origin. The increased trend toward globalization has adversely affected the current food scenario by the advertent importation of contaminated foods. The findings of the study suggest the need to develop strategies to reduce STEC in foods which will depend much on hygienic and sanitary production and processing practices. Thus, the colonization, transmission, and cross-contamination of STEC in foods and the environment can be minimized. An effective control measure for this pathogen has to target the farm, processing plants and the environments. At all these stages, strict adherence to standard operating measures must be practiced.

## Author’s Contributions

CS collected the samples, carried out the laboratory work. CL supervised the research work, reviewed the manuscript, and provided guidance for research work. ACJ contributed to the molecular work of the study and revised the manuscript. All authors read and approved the findings of the manuscript.
